# L‑RNA Aptamer-Based Tools for G‑Quadruplex Structure: Identification, Characterization, and Application

**DOI:** 10.1021/acs.accounts.5c00468

**Published:** 2025-09-30

**Authors:** Danyang Ji, Kun Zhang, Maryana Yarshova, Chun Kit Kwok

**Affiliations:** ¶ State Key Laboratory of Radiation Medicine and Protection, School of Radiation Medicine and Protection, Collaborative Innovation Center of Radiological Medicine of Jiangsu Higher Education Institutions, 12582 Soochow University, Suzhou 215123, China; ‡ Department of Chemistry and State Key Laboratory of Marine Environmental Health, 53025 City University of Hong Kong, Tat Chee Avenue, Kowloon Tong, Hong Kong SAR 999077, China; § Shenzhen Research Institute of City University of Hong Kong, Shenzhen 518057, China

## Abstract

Aptamers are single-stranded DNA or RNA oligonucleotides that bind specifically and strongly to their target molecules. However, the inherent instability of natural DNA and RNA aptamers in biological environments limits their applications. To overcome this limitation, we focused on the development and application of L-RNA aptamers composed of unnatural L-RNA nucleotides. The mirror stereochemistry of L-RNA confers enhanced stability against nuclease degradation, making it an ideal candidate for molecular targeting and biological applications. In addition, L-RNA’s inability to hybridize with D-DNA/RNA through Watson–Crick base pairing enables the selection of aptamers based on structure recognition. Our group focuses on targeting functional G-quadruplex (G4) structures that play critical roles in various cellular processes, including DNA replication, transcription, and translation, and are implicated in diseases such as cancers, neurological disorders, and viral pathogenesis.

This Account highlights our group’s recent efforts in developing novel and robust L-RNA aptamer selection platforms and tools for targeting functionally important G4 structures in different biological systems. Pioneering the L-RNA aptamer selection method for G4 structures, we have further established additional selection platforms enhancing SELEX (Systematic Evolution of Ligands by EXponential enrichment) efficiency, as well as binding affinity and specificity for G4 targets. Following lead aptamer identification and characterization using various biophysical and biochemical tools, our group has explored a number of innovative post-SELEX modification strategies to further improve the L-RNA aptamer’s functionality. Such include the development of circular L-RNA aptamers, L-RNA aptamer-antisense oligo (ASO) conjugates, L-RNA aptamer-fluorogenic RNA aptamer conjugates, and L-RNA aptamer-peptide conjugates for various *in vitro*, in-cell, and *in vivo* applications. From our studies, we have reported that these L-RNA aptamers can effectively regulate G4-mediated cellular processes by inhibiting G4-protein interactions and/or modulating transcription and translation and ultimately influencing gene expression and beyond.

Together, these works have advanced the accessibility, efficiency, and robustness of L-RNA aptamer technology, offering significant potential for the diagnosis and therapeutics of various diseases, particularly those related to G4 dysregulation. Our ongoing research seeks to further refine L-RNA aptamer selection, structure characterization, post-SELEX modification strategies, and their applications in biological and therapeutic contexts.

## Graphical abstract

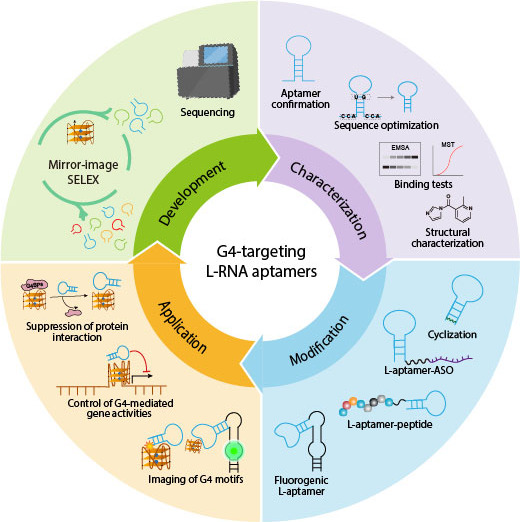

## Key references

- Ji, D.; Wang, B.; Lo, K. W.; Tsang, C. M.; Kwok, C. K. Predefined stem-loop structure library for discovery of L-RNA aptamers that target RNA G-quadruplexes. Angew. Chem. Int. Ed. 2025, 64, e202417247 10.1002/anie.20241724739462761.^1^ This work introduces G4-SLSELEX-Seq, a novel platform utilizing a predefined stem-loop structured library to enhance the efficiency of L-RNA aptamer selection against G4 targets.
- Zhao, H.; Lau, H. L.; Zhang, K.; Kwok, C. K. Selective recognition of RNA G-quadruplex in vitro and in cells by L-aptamer-D-oligonucleotide conjugate. Nucleic Acids Res. 2024, 52, 13544–13560 39558155 10.1093/nar/gkae1034PMC11662670.^2^ An L-RNA aptamer-ASO conjugate was engineered to achieve superior binding affinity and specificity toward the RNA G4 target in vitro and in cellular environments.
- Zhang, K.; Nie, Q.; Lau, C. K.; Kwok, C. K. Rational design of L-RNA aptamer-peptide conjugate for efficient cell uptake and G-quadruplex-mediated gene control. Angew. Chem. Int. Ed. 2024, 63, e202310798 10.1002/anie.20231079838156978.^3^ A rationally designed L-RNA aptamer-peptide conjugate is reported, demonstrating enhanced cellular uptake and enabling G4-mediated gene regulation.
- Ji, D.; Yuan, J.; Chen, S.; Tan, J.; Kwok, C. K. Selective targeting of parallel G-quadruplex structure using L-RNA aptamer. Nucleic Acids Res. 2023, 51, 11439–11452 37870474 10.1093/nar/gkad900PMC10681708.^4^ The G4-SELEX-Seq platform was developed, integrating high-throughput next generation sequencing to accelerate the selection of L-RNA aptamers targeting G4 structures.
- Umar, M. I.; Chan, C. Y.; Kwok, C. K. Development of RNA G-quadruplex (rG4)-targeting L-RNA aptamers by rG4-SELEX. Nat. Protoc. 2022, 17, 1385–1414 35444329 10.1038/s41596-022-00679-6.^5^ This protocol outlines the rG4-SELEX methodology for systematic generation of L-RNA aptamers targeting RNA G4.

## 1 Introduction

G-quadruplex (G4) motifs are noncanonical DNA or RNA structures that function as central modulators of genomic stability, gene regulation, and cellular homeostasis (Figure 1A).^6–8^ These structures arise from guanine-rich strands where planar guanine tetrads fold via Hoogsteen hydrogen bonds and are stabilized by monovalent cations; the stacking of tetrads is then facilitated by π–π interactions (Figure 1B). The resulting three-dimensional structure can adopt different topologies, the formation of which depends on the G-tract orientation as well as loop sequence and length, contributing to the overall thermodynamic stability of the structure.^9,10^ Formation at specific genomic/transcriptomic sites and protein interactions enable their participation within a wide range of biological processes like DNA replication, telomere, transcription, and translation regulation, while their dysregulation has been linked to various diseases, including cancer,^11^ neurological disorders,^12^ and viral pathogenesis,^13^ making them attractive therapeutic targets. However, G4’s structural versatility complicates their study and targeting due to their structural complexity, chemical composition, dynamic folding/unfolding behaviors, influence of environmental factors, and interacting partners. Despite this, their guanine-rich composition and unique bonding offer a distinct structure fingerprint for targeting compared to classical double helix DNA duplexes or other RNA structural motifs (e.g., hairpin loops or pseudoknots).

**Figure 1:**
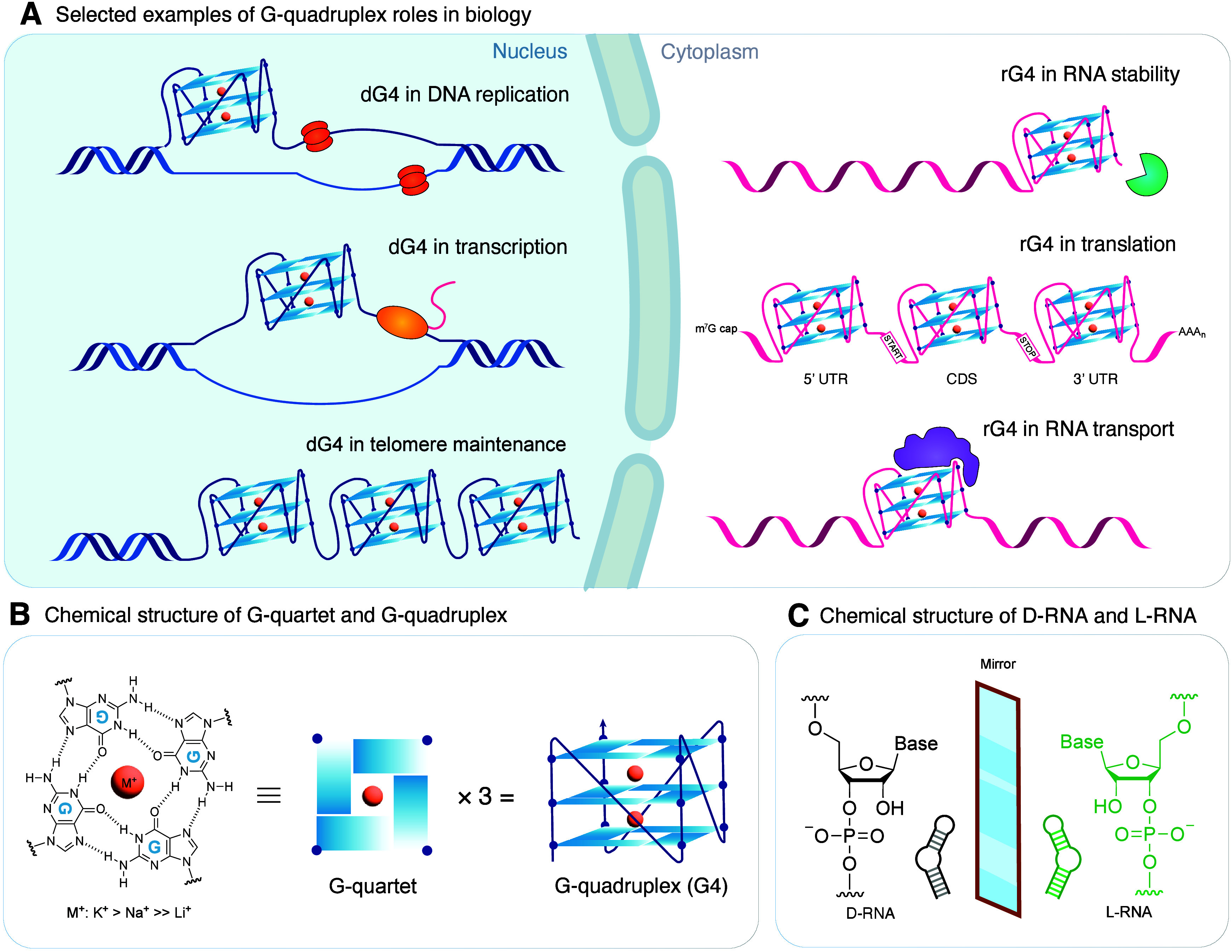
Biological role and structure of G-quadruplex (G4) and the chemical structure of D/L-RNA. (A) Selected examples of the roles of DNA G4s (dG4s) and RNA G4s (rG4s) in biology. (B) Chemical structure of the G-quartet and its subsequent folding into a G4 structure with parallel topology. G4 is stabilized by Hoogsteen hydrogen bonds and interactions with a monovalent cation (M^+^) with a preference of potassium (K^+^) > sodium (Na^+^) ≫ lithium (Li^+^). (C) Chemical structure of D-RNA as opposed to that of L-RNA, its synthetic enantiomer.

Current strategies to target G4s have employed small molecule ligands^14^ (e.g., 5,10,15,20-tetrakis­(*N*-methyl-4-pyridyl)­porphine (TMPyP4)^15^), fluorescent probes^16^ (e.g., thioflavin T (ThT),^17^ *N*-methyl mesoporphyrin IX (NMM)^18^), and antibodies (e.g., 1H6,^19^ BG4 ^20^), but some of these tools show limited selectivity between different G4 motifs or other nucleic acid structures. Certain small-molecule ligands, like TMPyP4, target G4s without distinguishing between DNA and RNA G4s^15,21,22^ and also bind to double helices.^23^ Fluorescent probes designed for G4 detection can suffer from binding to non-G4 nucleic acid structures, such as the benzothiazole fluorescent probe ThT, which binds tightly to G–A-rich sequences and may cause false positive and negative signals.^16,24^ Similarly, antibodies such as 1H6 exhibit cross-reactivity with thymidine-rich single-stranded DNA and denatured DNA fibers,^25^ thereby limiting their selectivity and reliability in identifying genuine G4 motifs. While these existing tools each offer distinct advantages, selectivity issues shared by some of these commonly used tools hinder identification and targeting of G4 motifs of interest as well as their potential application as therapeutics for G4-associated diseases. Consequently, this underscores the need for the development of an alternative method to study G4 structures.

Aptamers, which are single-stranded DNA or RNA oligonucleotides, offer a different molecule identification method and are selected through a process called SELEX (Systematic Evolution of Ligands by EXponential enrichment) for their high target binding affinity and selectivity provided by their specific three-dimensional structure and target-aptamer intermolecular interactions.^26–28^ Among these, L-RNA aptamers are synthetic nucleic acid molecules composed of L-RNA with inverted stereochemistry compared to natural D-RNA.^29,30^ This inverted stereochemistry provides resistance to nuclease degradation, overcoming the instability challenges of conventional D-DNA or D-RNA aptamers in biological environments (Figure 1C). It also prohibits Watson–Crick base-pairing formation with natural d-nucleic acids,^31^ making them a promising alternative for targeting higher-order nucleic acid structural elements, such as G4s, based on structural recognition.

This Account summarizes the evolution of L-RNA aptamer technology for G4 targeting developed by our group from novel SELEX platforms to aptamer characterization and modifications. We present applications for disrupting G4-protein interactions, regulating gene expression, and imaging G4s in cells. Finally, we highlight the current challenges and share our perspective on the future development of the field, including refining SELEX strategies, elucidating the 3D structures of L-RNA aptamers and their G4 target complexes, as well as addressing challenges in l-aptamer application *in vivo*.

## 2 Development of G4-targeting L-RNA aptamers

### 2.1 rG4-SELEX: The First L-RNA Aptamer Selection Platform Designed for G4 Targets

The generation of L-RNA aptamers relies on the “mirror-image” SELEX.^30^ In this strategy, a D-RNA aptamer is selected against the enantiomeric version of the desired target during the selection process. Once a high-affinity d-aptamer is identified, the corresponding l-aptamer is chemically synthesized. In 2020, our group introduced the application of L-RNA aptamers targeting G4 structures.^32,33^ We optimized the conventional mirror-image SELEX protocol for G4 targets, termed rG4-SELEX (Figure 2A).^5^ Modifications included adjusting SELEX conditions to accommodate G4 stability, such as optimizing the D-RNA library concentration, target L-G4 concentration per round, selection time for negative and positive selection, washing conditions, Mg^2+^ concentration, and incubation temperature. We used K^+^-containing buffer due to its physiological relevance for intracellular conditions and the stabilization of G4 structures.

**Figure 2:**
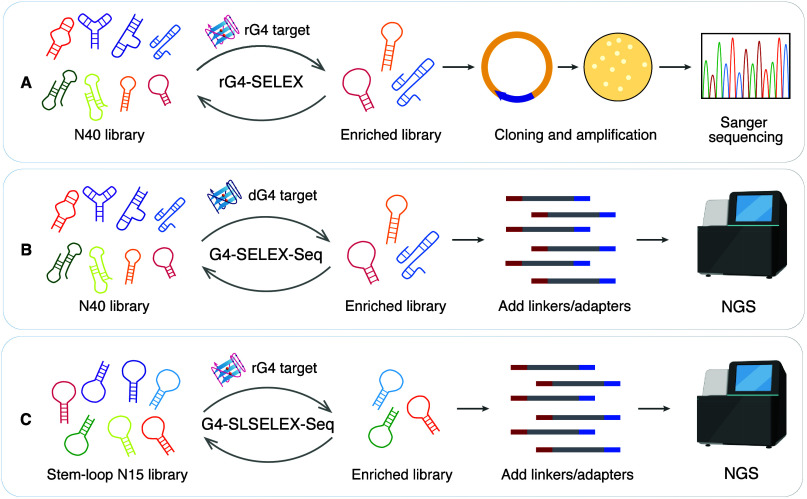
Platforms for developing G4-targeting L-RNA aptamers: (A) rG4-SELEX, the first L-RNA aptamer SELEX platform designed for G4 targets, utilizing low-throughput Sanger sequencing; (B) G4-SELEX-Seq, an advanced SELEX platform incorporating high-throughput NGS, improving both selection efficiency and success rate; (C) G4-SLSELEX-Seq, an optimized SELEX approach employing a predefined stem-loop structure library, significantly improving the selection efficiency for G4 targets.

Human telomerase RNA component (*hTERC*) rG4 was used to illustrate the rG4-SELEX workflow.^32^ The process began with a single-stranded DNA (ssDNA) library containing a 40-nucleotide randomized region (N40), which was converted into double-stranded DNA (dsDNA) via template extension and subsequently transcribed into a D-RNA library. After negative selection with tRNA-blocked streptavidin beads to remove sequences binding to beads, unbound D-RNAs underwent positive selection with biotinylated L-hTERC rG4. Target-aptamer complexes were captured on tRNA-blocked streptavidin beads, and bound D-RNAs were eluted, reverse-transcribed to cDNA, PCR-amplified, and used for subsequent rounds. After seven rounds, TA cloning and plasmid extraction were performed for Sanger sequencing. Using rG4-SELEX, we successfully identified three high-affinity L-RNA aptamers targeting *hTERC* rG4, *TERRA* rG4, and *APP* rG4, demonstrating the platform’s efficiency.^32–34^

### 2.2 G4-SELEX-Seq: Utilizing High-Throughput NGS to Improve Selection Efficiency

Despite initial failures to adapt rG4-SELEX for DNA G4 (dG4) targets, even after extending the selection to 7–15 rounds, Sanger sequencing data revealed several enriched sequences across different dG4 targets. These nonspecific enriched sequences dominated the pool, accounting for over 80% of the sequenced clones, potentially arising from enzyme bias or nonspecific interactions during the selection process. While similar nonspecific sequences appeared in previous rG4 targets, they occurred at much lower frequencies. This discrepancy suggested that rG4s, due to their structural flexibility and greater propensity for hydrogen bonding, facilitate stronger and more specific interactions with aptamers, enabling genuine binders to outcompete nonspecific enriched sequences. In contrast, dG4s may engage in weaker interactions, allowing nonspecific sequences to dominate the selection process.

To address this issue, we proposed utilizing high-throughput next-generation sequencing (NGS) to analyze SELEX results instead of low-throughput Sanger sequencing (Figure 2B),^4^ allowing comprehensive sequence information to be obtained for each round. We initially applied NGS to the *c-kit* dG4 target, conducting sequencing after four selection rounds.^4^ The results revealed that two highly enriched sequences exhibited dramatic increases in abundance throughout the selection process, which also appeared in unrelated SELEX experiments. By disregarding the nonspecific sequences and focusing on the remaining candidates, we identified Apt12 as the strongest binder through binding validation. This approach enabled the identification of a binding sequence for *c-kit* dG4 within four selection rounds, significantly enhancing both the success rate and selection efficiency compared to traditional Sanger sequencing. We termed this NGS-based method as G4-SELEX-Seq, which has been shown to be applicable not only for DNA G4 target^4^ but also RNA G4 targets, facilitating the successful identification of binding aptamers for *HIV-1* rG4,^35^ *APP* rG4,^36^ and pUG fold RNA.^37^

### 2.3 G4-SLSELEX-Seq: Employing a Stem-Loop Structured Library to Accelerate L-RNA Aptamer Discovery for G4 Targets

Although G4-SELEX-Seq improved the selection efficiency for G4-binding L-RNA aptamers, it still required multiple rounds (often seven or more) for several targets due to the low initial abundance of functional sequences in the standard N40 library. Further optimization to the SELEX platform was needed due to the platform selecting aptamers primarily being based on binding affinity, overlooking binding specificity. A structural analysis of previously identified G4-binding L-RNA aptamers revealed a common stem-loop (SL) architecture with loop nucleotides mediating G4 target recognition and stem structure providing stability.^4,32–34^ We hypothesized that prestructuring the initial library with stem-loop elements could bias the selection toward functional G4 binders. This led to the development of G4-SLSELEX-Seq, which employs a predefined SL structured library with a 15-nucleotide randomized region (N15) in the loop (Figure 2C).^1^ Moreover, we incorporated an additional negative selection step in G4-SLSELEX-Seq to eliminate the sequences binding to nontarget G4s, improving the stringency and enhancing binding specificity of the selection process.

By employing G4-SLSELEX-Seq, we identified a binding aptamer for *EBNA1* rG4 within three selection rounds.^1^ To validate the reliability and versatility of this method, we applied the G4-SLSELEX-Seq platform to two additional G4 targets, *APP* rG4 and *HCV-1a* rG4, and similarly identified high-affinity aptamers for both in three rounds.^1^ These results highlighted that G4-SLSELEX-Seq significantly accelerates the discovery of G4-targeting L-RNA aptamers while maintaining high specificity and binding affinity.

In short, our studies have established a systematic pipeline for the development of G4-targeting L-RNA aptamers, beginning with the rG4-SELEX platform for RNA G4s, followed by G4-SELEX-Seq to overcome challenges in DNA G4 selections and culminating in G4-SLSELEX-Seq, which leverages structural insights to maximize efficiency. A comprehensive list of L-RNA aptamers developed by the Kwok lab, with their targets, selection platforms, and characteristics, is provided in Table S1 of the Supporting Information. *In vitro* selection platforms established by our group are not limited to G-quadruplexes, but provide a framework for discovering l-aptamers against other structured nucleic acid motifs. By tailoring selection parameters, including buffer conditions, cations, pH, and target structures, this approach can be tailored to other functionally significant motifs, such as pseudoknots, triplexes, i-motifs, and others.

## 3 Characterization of G4-Binding L-RNA Aptamers

### 3.1 Aptamer Candidate Confirmation and Sequence Optimization

Following iterative selection rounds, potential aptamer candidates are identified from sequencing data (Figure 3A). Our group typically processes 20–30 clones for Sanger sequencing to identify enriched sequences for downstream validation. For NGS, candidates must be identified from millions of sequences ranking them based on their abundance. Sequences dominating in early rounds with disproportionate enrichment are typically disregarded as nonspecific enrichment arising from PCR bias or nonspecific interactions. For the remaining candidates, conservation across rounds is assessed with well-conserved, high-frequency sequences prioritized for further evaluation. Bioinformatics tools like FASTAptamer and AptaSUITE provide insights into data quality, sequence frequency, mutational patterns, and conserved motifs.^38,39^ AptaSUITE further enables clustering based on their predicted secondary structures.^39^ After identifying potential aptamer candidates, we use Mfold to predict the secondary structures of full-length (N40 plus primer binding domains, 78 nt) and the N40 versions of the candidates.^40^ To ensure that binding interactions are involved in the N40 region only rather than primer-binding domains, we select sequences whose N40 regions maintain a consistent structural integrity with or without the primer binding region. Initial binding affinity and specificity tests are then conducted to identify the most promising candidates.

**Figure 3:**
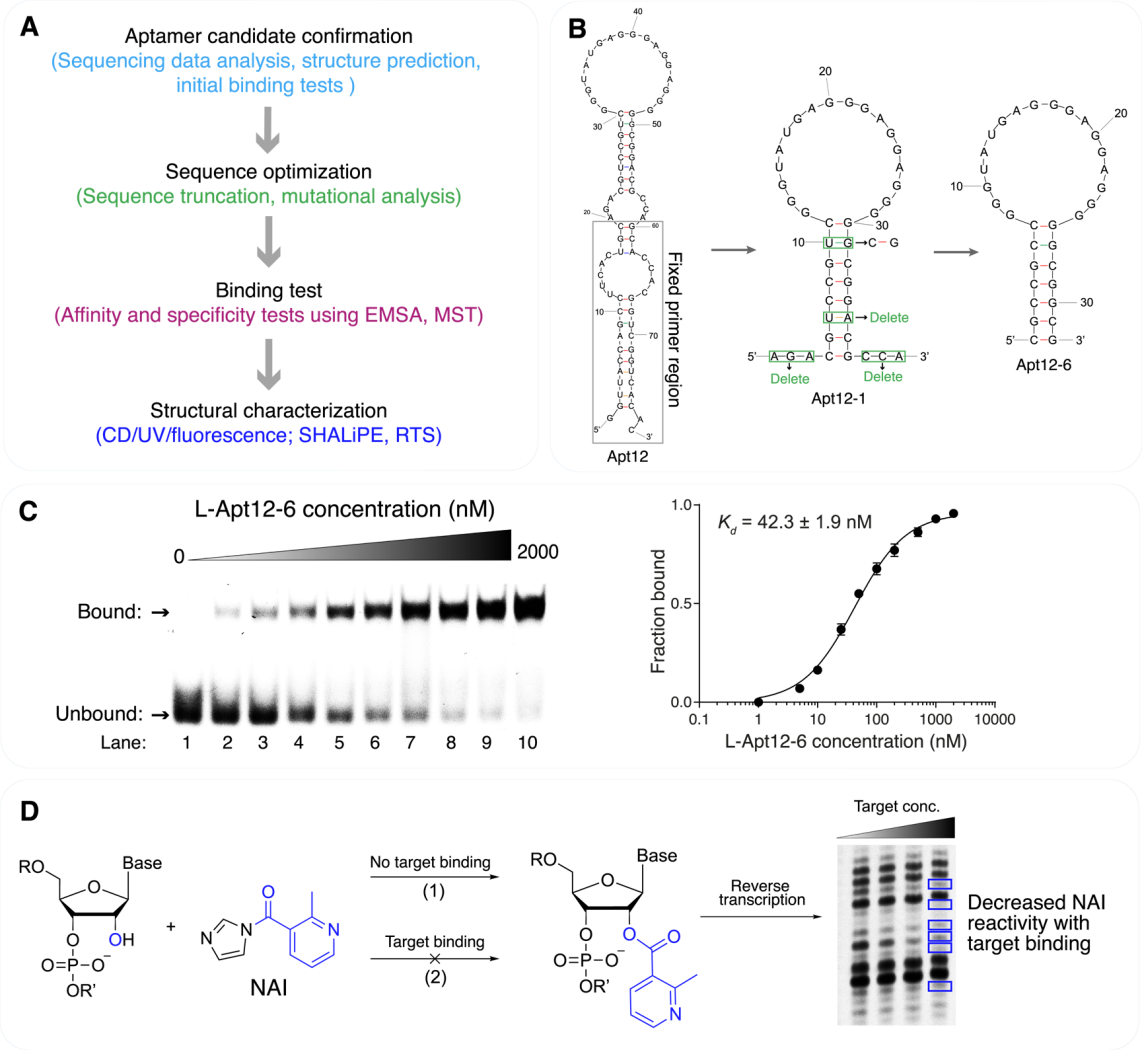
Flowchart and representative example of the characterization of G4-binding L-RNA aptamers. (A) Aptamer characterization process includes candidate confirmation, sequence optimization, binding tests, and structural characterization. (B) Using Apt12 as a representative case, in the sequence optimization step, the fixed primer region of full-length aptamer (Apt12) for G4-SELEX-Seq is boxed in gray. The N40 region (Apt12-1) was truncated by deleting the two flanking ends (5′-AGA and 3′-CCA) and the “U–A” base pair in the stem. To strengthen the stem region, the “U–G” base pair originally in the stem region of Apt12-1 was mutated to “C–G” and resulted in the final 32-nt aptamer sequence Apt12-6. Adapted with permission from ref 4. Copyright 2023, Published by Oxford University Press on behalf of Nucleic Acids Research. (C) EMSA shows the binding between L-Apt12-6 and FAM-D-c-*kit* 1 dG4. *K*_d_ was calculated as 42.3 ± 1.9 nM based on the EMSA results. Adapted with permission from ref 4. Copyright 2023, Published by Oxford University Press on behalf of Nucleic Acids Research. (D) In the SHALiPE assay, NAI modifies flexible ribonucleotides in the aptamer sequence and causes stalling in the reverse transcription gel. The direct interaction of ribonucleotides with G4 targets protects them from NAI modification and causes decreased NAI reactivity for interaction sites. The gel image was adapted with permission from ref 4. Copyright 2023, Published by Oxford University Press on behalf of Nucleic Acids Research.

Once a lead aptamer candidate is confirmed, we proceeded with sequence truncation to optimize aptamer length and binding. Since G4-targeting L-RNA aptamers predominantly adopt stem-loop structures, optimization begins with removing nonessential flanking regions from 5′ and 3′ ends. While stems typically do not directly engage the target, their length and rigidity critically affect binding performance. Longer stems provide enhanced structural stability, rigidifying loop conformation, and L-aptamer architecture to improve target binding. To minimize aptamer size while maintaining binding, we reduced stem length to essential base pairs for structural integrity (Figure 3B).^4^ Single-nucleotide mutagenesis of loop regions identified base substitutions enhancing binding, revealing that key loop residues govern binding affinity. Binding assays then verify that truncated regions do not affect binding. This optimization process yielded the final, minimized D-RNA aptamer for L-RNA synthesis and downstream studies.

### 3.2 Binding Affinity and Specificity Tests

Using the L-RNA aptamer, we conduct binding tests, including affinity and specificity evaluations. The electrophoretic mobility shift assay (EMSA) is the primary method employed for assessing interactions between L-RNA aptamers and G4 targets. This method offers clear visual results and requires simple instrumentation. In the EMSA, the G4 target is fluorescently labeled (e.g., FAM or Cy5) for gel imaging. The resulting target-aptamer complex exhibits reduced mobility in the gel, allowing for visual identification of a gel shift (Figure 3C).^4^ Dissociation constants (*K*_d_) are calculated from EMSA data to quantify binding affinity, with lower *K*_d_ values indicating stronger interactions (Figure 3C). Microscale thermophoresis (MST) provides orthogonal validation of target–aptamer interactions by detecting changes in molecular movement under a microscopic temperature gradient.^4^ Specificity is assessed against a panel of nontarget dG4s, rG4s, and non-G4 nucleic acids to confirm selective recognition of the intended G4 structure. Chirality-dependent binding is verified by testing interactions between: L-aptamer and L-target (negative control), D-aptamer and D-target (negative control), D-aptamer and L-target (binding expected), and L-aptamer and D-target (binding expected). The absence of binding in homochiral (L-L or D-D) pairs and robust binding in heterochiral (D-L or L-D) pairs confirm cross-chirality recognition, a hallmark of mirror-image aptamers.

### 3.3 Structural Characterization

Structural characterization is conducted to gain deeper insights into the interactions between the target G4 and L-RNA aptamers. As L-RNA aptamers may contain G4 motifs that could interfere with structural characterization, we first assess the G4-forming potential of G-rich sequences using established prediction tools, such as QGRS,^41^ G4Hunter,^42^ and G4NN.^43^ Then, the aptamers are evaluated by biophysical experiments: Circular dichroism (CD) spectroscopy determines the overall conformation of aptamers by measuring the differential absorption of left- and right-handed circularly polarized light and determine G4 topology (e.g., parallel, antiparallel) via characteristic spectral peaks; UV melting assays measure thermal stability of aptamers by monitoring absorbance changes at 260 or 295 nm (for G4-containing aptamers); fluorescence-based assays employ G4-specific probes (e.g., NMM) to confirm G4 formation.^4^ SHALiPE (selective 2′-hydroxyl acylation analyzed lithium ion-mediated primer extension) and reverse transcriptase stalling (RTS) assays are used to study aptamer structure in single nucleotide-resolution using 2-methylnicotinic acid imidazolide (NAI).^4,44^ NAI can selectively modify flexible ribonucleotides in ssRNA regions and block reverse transcriptase elongation, resulting in an NAI modification pattern in denaturing gel.^4^ In SHALiPE, the D-RNA aptamer is incubated with increasing concentrations of the L-G4 target first and then modified by NAI, followed by primer extension to identify ribonucleotides whose reactivity or stalling patterns change upon target binding, indicative of potential interaction sites (Figure 3D). While biochemical and biophysical assays provide critical insights into aptamer-G4 interactions, high-resolution structural determination remains essential for elucidating precise binding mechanisms.

## 4 Post-SELEX Modification of G4-Binding L-RNA Aptamers

### 4.1 Circular L-RNA Aptamers Enhance Stability and Affinity

l-Aptamers exhibit superior biostability compared to natural d-aptamers due to nuclease degradation resistance.^1,4,32–34^ Physiochemical factors like pH, temperature, and salt concentration can significantly affect aptamer conformation and functionality.^45^ Circular nucleic acids often exhibit enhanced thermal and conformational stability.^46,47^ Among chemical and enzymatic cyclization methods, click chemistry offers a straightforward approach for ligating nucleic acids.^48–50^ To improve the conformational stability and binding performance of L-RNA aptamers in complex conditions, we developed the first circular L-RNA aptamer, cycL-Apt.4-1c. We rationally designed 2 linkers modified with an alkyne and azide residues at the 5′ and 3′ terminals of linear L-Apt4-1c and cyclized it through an efficient copper­(I)-catalyzed azide–alkyne cycloaddition (CuAAC) reaction (Figure 4A).^51^ Circular L-RNA aptamer (cycL-Apt.4-1c) showed greater conformational stability under urea-mediated denaturing conditions. It exhibited a 10-fold improved binding affinity and specificity to the G4 target, expanding its applications in inhibiting RNA-protein interactions and gene regulation activities.

**Figure 4:**
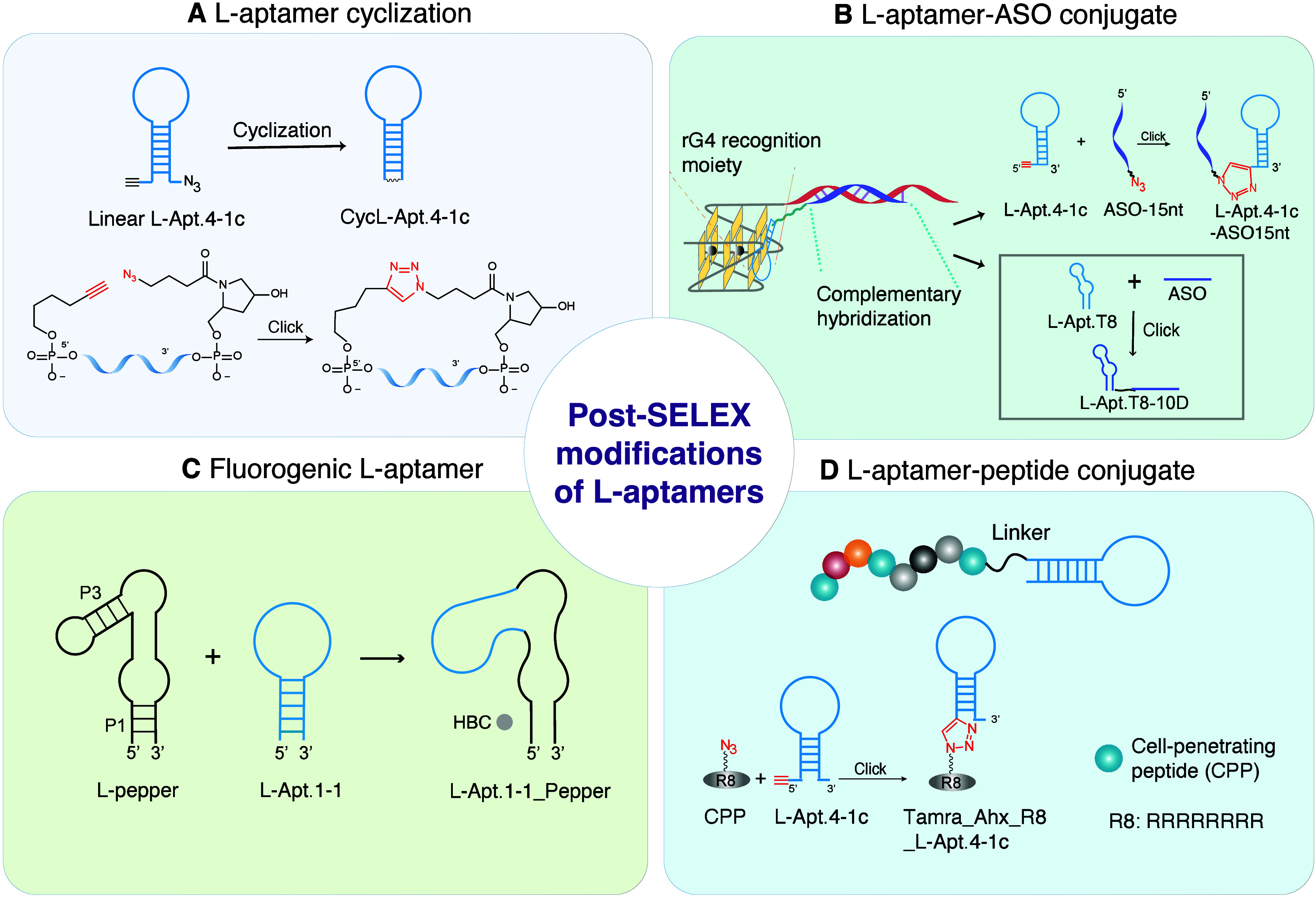
Representative post-SELEX modifications of l-aptamers. (A) L-RNA aptamer cyclization to reduce conformational flexibility and improve target binding affinity through an intramolecular CuAAC reaction. CuAAC: Copper­(I)-Catalyzed Azide–Alkyne Cycloaddition. cycL-Apt.4-1c: cyclized L-RNA aptamer. Adapted with permission from ref 51. Copyright 2021, Published by Oxford University Press on behalf of Nucleic Acids Research. (B) Design of l-aptamer–antisense oligonucleotide (ASO) conjugates aimed at recognizing specific rG4 regions of interest. The l-aptamer segment recognizes the rG4 motif, while the ASO component identifies the flanking sequence near the rG4 motif through sequence specificity. L-Apt.T8-10D: L-Apt.T8-10nt ASO. L-Apt.4-1c-ASO15nt: l-aptamer-15nt ASO. (C) Development of a bifunctional aptamer by fusing L-Apt1.1 to the P3 region of the l-pepper aptamer. l-Pepper aptamer, the enantiomeric form of d-pepper aptamer, is one of the fluorogenic RNAs that binds to HBC ligands and activates their corresponding fluorophores. (D) L-RNA aptamer-CPP conjugate for efficient cell uptake. CPP: Cell-Penetrating Peptide.

### 4.2 L-RNA Aptamer-Antisense Oligo (ASO) Conjugates Enhance Specificity and Affinity

Due to G4s’ structural similarity, distinguishing specific rG4s remains challenging. To achieve individual G4 specificity, we engineered L-RNA aptamers with sequence-specific DNA ASOs. Using L-Apt.4-1c as an example, we developed the first l-aptamer-ASO conjugate, L-Apt.4-1c-ASO15nt­(APP), able to specifically recognize the *APP* rG4 region *in vitro* and in cells. This conjugation strategy enhances the affinity of the L-Apt.4-1c toward the *APP* G4 target by 150-fold.^2^ Another l-aptamer, L-Apt.T8-10D, which incorporates a 10-nt DNA antisense (10D), has shown improvements in both binding affinity and selectivity compared to the original L-Apt.T8 (Figure 4B).^35^ Overall, the coupling of L-RNA aptamers with ASO represents an advancement in the design of selective and effective molecular tools for G4-targeting.

### 4.3 L-RNA Aptamer-Fluorogenic RNA Conjugates Achieve G4 Imaging

Using L-RNA aptamers to visualize G4 structures requires careful consideration of factors such as specificity, binding affinity, and the biological context of the interactions being observed.^52^ Recently, we developed a novel non-G4-containing l-aptamer, L-Apt.1-1, with nanomolar binding affinity and improved binding specificity to *APP* rG4. Pepper, a fluorogenic RNA aptamer that binds to a diverse range of benzylidene-cyanophenyl (HBC) derivatives, has been demonstrated to have significant potential in the detection of RNA molecules.^53^ By introducing L-Apt.1-1 to the P3 stem of l-pepper aptamer, the enantiomeric form of d-pepper aptamer, we designed the first L-RNA-based bifunctional aptamer, L-Apt.1-1_Pepper. This system can achieve *APP* rG4 target-dependent fluorescent light-up with the highest fold of 47.8 ± 3.7. Notably, L-Apt.1-1_Pepper aptamer takes advantage of high stability of l-oligonucleotides in the cellular environment, indicating that it can be applied to perform cellular imaging of *APP* rG4 (Figure 4C).^36^

### 4.4 L-RNA Aptamer-Peptide Conjugates Improve Cellular Penetration

Despite frequent penetration in cellular membranes, L-RNA aptamers showed improved translocation efficiency when attached to cell-penetrating peptides (CPPs), without the need for transfection reagent (Figure 4D).^3^ The l-aptamer-peptide conjugate Tamra_Ahx_R8_L-Apt.4-1c efficiently enters cells and targets rG4 in various mRNA regions (3′UTR and 5′UTR). This improved cellular uptake enhances the aptamer’s therapeutic efficacy and allows for more versatile applications in gene regulation, providing a robust platform for developing targeted therapies.

## 5 Applications of L-RNA Aptamer-Based Tools

### 5.1 Modified L-RNA Aptamers Suppress G4-Protein Interactions

G4s interact with various proteins that can either stabilize or destabilize their formation, influencing downstream signaling pathways and gene expression.^54^ The ability of L-RNA aptamers to inhibit G4-protein interactions is a critical application in the field of molecular biology. Linear and cyclic L-Apt.4-1c have been developed to bind specifically to the rG4 structure of the *hTERC* long noncoding RNA (lncRNA), which is known to be involved in telomerase activity and cellular proliferation.^51^ These aptamers bind preferentially to rG4s over non-G4s and effectively inhibit the interaction between rG4 and RHAU-specific motif-containing 53 amino acids (RHAU53, the truncated DHX36 fragment) or full-length DHX36 protein.^55^ Linear L-Apt.4-1c also interfered with the *hTERC* rG4-nucleolin interaction, a protein involved in many cellular pathways across the nucleus and cytoplasm.^32,56^ Also, we demonstrated the formation of the rG4 motif in *MALAT1* lncRNA and the interaction between *MALAT1* rG4 and NONO protein can be disrupted by linear L-Apt.4-1c (Figure 5A).^57^ These inhibitions disrupt the normal function of the G4-protein complex and provide insights into the regulatory mechanisms governing G4 dynamics within the cell.

**Figure 5:**
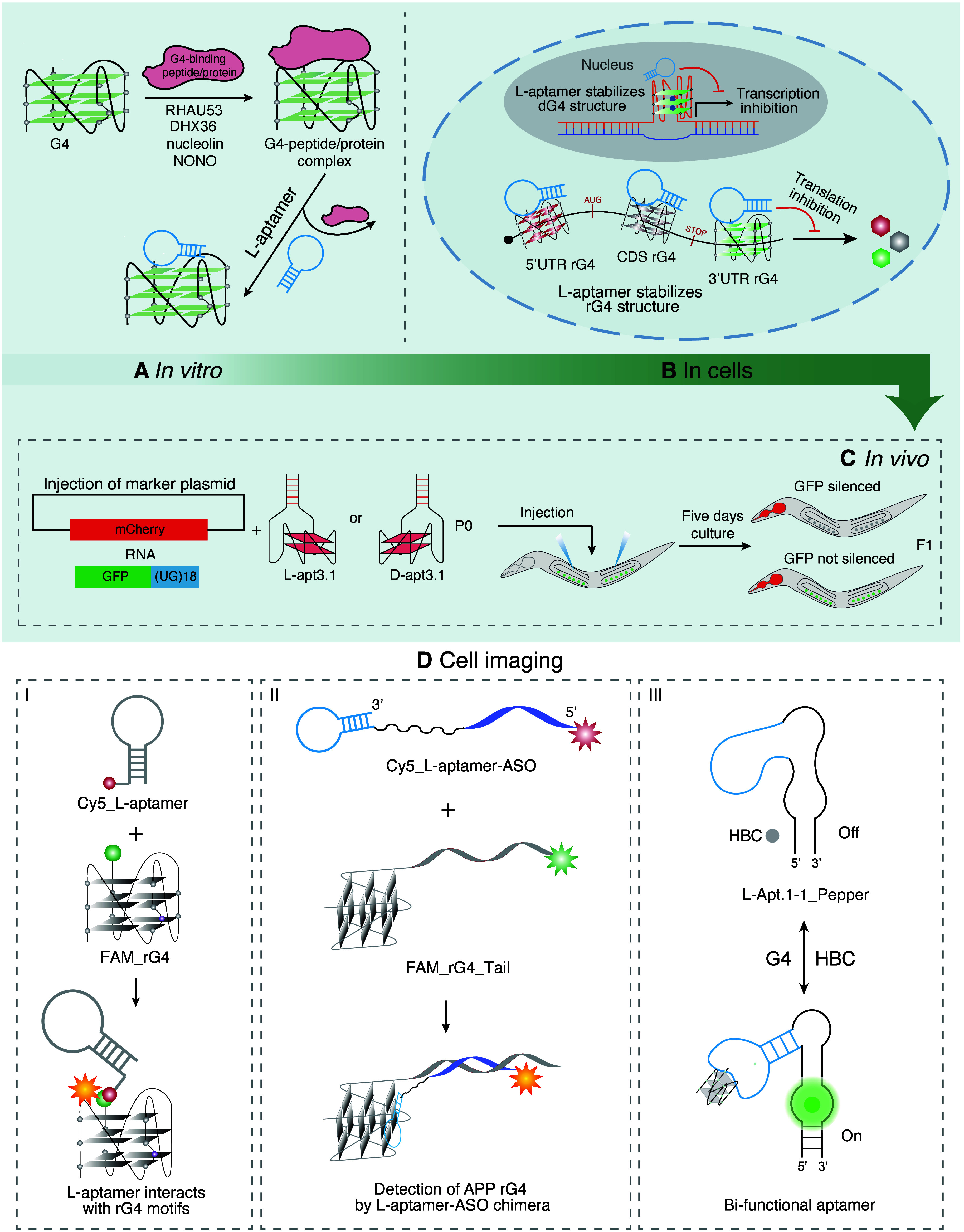
Applications of representative modified l-aptamers. (A) l-Aptamers disrupt the interaction between rG4s and G4 binding peptides/proteins. DHX36: DEAH-Box helicase36. RHAU53: RHAU-specific motif-containing 53 amino acids. (B) l-Aptamer mechanism for gene transcription and translation regulation. l-Aptamers can suppress transcriptional and post-transcriptional gene expression by regulating the rG4 motif in the promoter domain and different mRNA regions (5′UTR, CDS, 3′UTR). (C) l-Aptamer regulates gene expression *in vivo*. The marker plasmid and GFP pUG RNA were microinjected with the indicated molecules (L-apt3.1, D-apt3.1). L-apt3.1 could interact with poly­(UG) (pUG) fold and inhibit pUG RNA’s function in *C. elegans*. Adapted with permission from ref 37. Copyright 2025, Published by Oxford University Press on behalf of Nucleic Acids Research. (D) The diverse applications of l-aptamers in cell-imaging assays. I and II: FAM-rG4s/FAM-rG4s_Tail and Cy5_L-aptamers/Cy5_L-aptamers_ASO were transfected into cells to analyze colocalization and monitor specific interactions between them. III: L-Apt.1.1_Pepper sensor was developed to achieve specific fluorescence light-up against *APP* rG4 by binding to both HBC530 ligand and *APP* rG4 in cells.

### 5.2 Modified L-RNA Aptamers Control G4-Mediated Gene Activity

The capability of L-RNA aptamers to influence G4 gene activity offers a promising therapeutic approach, especially in diseases where G4 structures play a crucial role, such as neurodegenerative disorders and cancers.^11,12^ By targeting specific G4 structures within different regions of mRNA (5′UTR, Coding Sequence CDS, and 3′UTR), modified L-RNA aptamers can influence translational activity and thus regulate gene expression profiles. First, cycL-Apt.4-1c and Tamra_Ahx_R8_L-Apt.4-1c have shown the negative regulating roles on the human *hTERC* and *NRAS* 5′UTR rG4s in a dual-luciferase reporter assay, respectively.^3,51^ Furthermore, recent studies have highlighted the role of 3′UTR rG4s in regulating the expression of proteins associated with neurodegeneration, including the amyloid precursor protein (APP).^58^ A novel L-RNA aptamer, L-Apt.8f, binds to *APP* 3′UTR rG4 strongly and controls the *APP* reporter gene and native transcript translation in cells. Also, developing non-G4-containing L-RNA aptamers, L-Apt.1-1, has demonstrated a strong binding affinity to *APP* rG4s. It can effectively regulate *APP* gene expression by stabilizing the rG4 structure, leading to translation repression.^36^ More recently, the development of L-RNA aptamer-antisense oligonucleotide conjugates, L-Apt.4-1c-ASO15nt (APP), has shown promise in enhancing the specificity and precise modulation of endogenous *APP* gene expression in neuronal cells.^2^ Finally, we have demonstrated that L-RNA aptamers can effectively downregulate the expression of the Epstein–Barr nuclear antigen 1 (EBNA1) by targeting CDS rG4 in EBV-positive cancer cells, showcasing their capacity for the selective inhibitory effect on virus-associated cell growth.^1^

The influence of L-RNA aptamers on transcriptional activity is crucial for gene regulation. G4 structures, prevalent in many oncogenes , represent a strategic target for L-RNA aptamers.^6,8,59^ By binding to these structures, L-RNA aptamers can disrupt normal gene function, leading to reduced expression of transcripts and proteins that drive disease pathology. Studies have shown that L-Apt.12-6 can be engineered to bind to G4s in the promoter domain of the human proto-oncogene *c-KIT*, leading to transcriptional repression and subsequent inhibition of c-KIT protein expression.^4^ This regulatory capability highlights the potential of L-RNA aptamers as therapeutic agents in cancer treatment and can be used to silence overexpressed oncogenes (Figure 5B).

More recently, we have expanded the application of L-RNA aptamers *in vivo*. The pUG fold RNA, a unique RNA structure characterized by its poly­(UG) composition, plays a significant role in gene regulation and silencing mechanisms. This RNA structure is particularly notable in the model organism *Caenorhabditis elegans*, where it is involved in RNA interference (RNAi) pathways.^60^ The pUG fold RNA has been shown to adopt a quadruplex (G4) structure that is essential for its biological functions, including the recruitment of RNA-dependent RNA polymerase (RdRP), which synthesizes small interfering RNAs that mediate gene silencing.^61^ Recently, L-apt3.1 has been developed to bind strongly to the pUG fold and inhibit gene silencing in *Caenorhabditis elegans*.^37^ Notably, the ability of L-RNA aptamers to interact with pUG folds *in vivo* suggests the potential of l-aptamers for controlling gene expression in organisms, paving the way for diverse applications in different cellular systems, as well as *in vivo* settings (Figure 5C).

### 5.3 G4 Imaging Using Modified L-RNA Aptamers

Developing imaging techniques for G4 structures is crucial for understanding their biological roles and dynamics.^6^ High-affinity interactions, as demonstrated by aptamers like L-Apt.8f and L-Apt.4-1c-ASO15nt­(APP), enable robust imaging signals that facilitate the detection of *APP* rG4s in complex cellular environments.^2^ Additionally, the development of fluorogenic L-RNA aptamers enables real-time imaging of G4s, providing insights into their dynamics and spatial distribution in living cells. L-Apt.1-1_Pepper aptamer, engineered as a light-up probe, allows for specific cellular imaging of the *APP* rG4 structure (Figure 5D).^34,36^ This imaging capability not only enhances our understanding of G4 biology but also opens avenues for the biosensing and bioimaging of G4s.

## 6 Summary and Outlook

L-RNA aptamers are a new class of G4-targeting tools with great potential. This Account highlights progress made by our group in the development of aptamer selection platforms, exploration of aptamer structure and target–aptamer interaction, diverse post-SELEX modifications, and applications for regulating G4-mediated biology. Looking ahead, several key directions will shape the future of this research area.

Continued advancement in SELEX technology will be critical to improving the affinity, specificity, and other molecular properties of the selected aptamers. Several novel SELEX- and non-SELEX-based platforms have been developed recently to generate aptamer for diverse protein/small molecule targets, and these innovations can likely be adapted to the mirror image SELEX described above.^62–68^ With the rational design of library and aptamer selection criteria, we have showcased that both G4-containing and non-G4-containing L-RNA aptamers can be developed to recognize the G4 target of interest.^36,52^ For cellular application, future L-RNA aptamer selection may prioritize non-G-rich libraries, as selected L-RNA aptamers with single-stranded G-rich regions were recently shown to be more cytotoxic than their D-DNA/RNA counterparts.^69^

Second, resolving the 3D structure of the L-RNA aptamer-D-G4 target complex remains paramount. As of now, no high-resolution structure of the L-RNA aptamer-D-G4 target has been reported. The conformational dynamics and structural flexibility of L-RNA aptamers and G4 targets hinder the formation of homogeneous complexes for high-resolution analysis. Furthermore, typically low yields and high cost of chemically synthesized L-RNA have limited the material for structural studies. To overcome these obstacles, we advocate for the advancement of specialized high-resolution methods including include cryo-electron microscopy (cryo-EM) aided by scaffolding or oligomerization strategies,^70^ solid-phase synthesis of aptamer and/or target incorporating isotopically labeled phosphoramidites to resolve spectral overlap in NMR studies,^71^ and chaperone-assisted RNA X-ray crystallography.^72^ Understanding these structures will guide the rational design of enhanced aptamers and help build a database for AI-powered structure prediction of l-form nucleic acids.

The main advantage of L-RNA aptamers lies in their resistance to degradation by nucleases and the ability to recognize targets with high affinity and specificity based on structural recognition. Nevertheless, achieving high affinity and specificity in complex biological fluids is challenging due to low target concentration and interference from biological matrices, unlike *in vitro* conditions, with the influence of inverse chirality on aptamer behavior not being extensively studied.^73^ Moreover, G4-targeting L-RNA aptamers can influence translational activity by targeting G4 structures in mRNA regions (5′ UTR, coding sequence (CDS), and 3′UTR), indicating their ability to stabilize G4 formation in cells. Future studies may investigate G4 folding equilibrium through quantitative methods like surface plasmon resonance (SPR), single-molecule FRET microscopy, and circular dichroism (CD) spectroscopy, compared with other G4 ligand toolsets. To enable L-RNA aptamer adoption as diagnostic or therapeutic tools, further research must characterize *in vivo* pharmacokinetics of L-RNA aptamers and mitigate unintended immunogenicity, cytotoxicity, and off-target effects by taking inspiration from the d-aptamer field, including conjugation strategies, sequence optimization, and delivery systems tailored for l-aptamers.^74,75^

Considering the demonstrated potential of L-RNA aptamers in the identification and characterization of G4s, as well as their application in G4 biology, we are positive that these L-RNA aptamer-based tools can further deepen our understanding of the biological role of G4s and possibly other nucleic acid structure motifs in health and disease.

## Supplementary Material



## Notes

Table S1: Summary of G-quadruplex-targeting L-RNA aptamers (XLSX)
